# Traveling Letter, No. VII

**Published:** 1844-11

**Authors:** 


### THE WESTERN JOURNAL.

New Series.—Vol. II.—No. V.

LOUISVILLE, NOVEMBER 1, 1844.

### TRAVELING LETTERS FROM THE SENIOR EDITOR.

### NO. VII.

Peoria, Illinois, September 17, 1844.

*To my Colleagues:*

Gentlemen:—On my way to this place, through several towns of this State, I heard much of the

*Epidemic Erysipelas,*

but saw no case, at least none that was well characterized. It prevailed last winter and spring, but like scarlatina, passed by many localities, while it fell heavily on others. Believing that our readers would bear another communication from me on the experience of the profession in this quarter, I was about to make one, when a newspaper containing an account of the disease, as it appeared and was treated in and around Bloomington, M'Lean county, east of this place, fell into my hands, and will be found better for our readers, than any thing I could serve up for them. It embodies the experience of our friend Dr. John F. Henry, a sound practitioner and most candid narrator, and therefore merits a more permanent place than that of a village newspaper. I send you the paper and request its insertion at the close of this letter.

Since I left the Illinois river at Mendocia, a fortnight since, and proceeded by way of Jacksonville, Springfield, and Bloomington to this place, I have every where met with

*Autumnal Fever*, which prevails as much as it has ever done before, but in general is a mild tertian or double tertian. Now and then a malignant case presents itself, the collapse in which is as fatal as was that of epidemic cholera, and quite as easily averted oy timely and appropriate medication. The disease is by no means confined to the water-courses, but occurs in the depths of the high rolling praries.

The meridian in which I now am, does not more abound in autumnal fever, than in

*Medical Schools.*

In St. Louis there are two, in Jacksonville one, and in Chicago another, making four, all commenced within as many years, in a region which a short time since was the Far West.

The oldest is the *Medical Department of Kemper College*, St. Louis, projected by Professor McDowell, and known in the surrounding country as 'McDowell's School,' that gentleman being at once its Achilles and Ulysses. It has a respectable edifice, and its last class amounted, I understand, to 60, chiefly practitioners and students from Missouri and Illinois.

The next in age, is the *Medical Department of the University of St. Louis*, founded by Professors Hall and Prout, seceders from the last; but Professor Prout's withdrawal from this, also, leaves Professor Hall, in the undivided honor of being its projector. Less distinguished for public speaking than his rival, the Professor seems to possess a quiet performance, the fruits of which are the organization of a full Faculty, and the erection of a suitable edifice, which will be ready for occupation by the ensuing November. Its class last year was, if I recollect right, between 20 and 30.

I am acquainted with, and might say something, of a part of the Professors of each of these young colleges, but as 'I have not come,' either 'to praise Cæsar,' or 'to bury him,' I shall content myself with adding, that the care of the Hospital, is confided to the Faculty of one school for half the session, and to the other for the remainder; the pupils of each having equal access to it throughout the winter.

The next of which I shall speak, is the *Medical Department of Illinois College.* As I passed through Jacksonville, I made acquaintance with the only two of its Professors who reside in that town, Dr. Jones and Dr. Adams. The latter, who holds the chair of Chemistry, Materia Medica, and Therapeutics, is also Professor of Chemistry and Natural History, and teacher of Modern Languages in the Academical Department; Dr. Jones, Professor of Obstetrics, appears to have been the founder of the establishment. The other Professors, reside, I believe, in different places. Jacksonville has no hospital, but the faculty promises the advantages of a dispensary to their pupils. The class of last winter, according to the printed catalogue, numbered fourteen. Cost of tickets $60.

As I have not yet reached Chicago, I can only speak of its school from an advertisement, which, a few days since, I cut out of a newspaper. Its title is the *Rush Medical College,* its Professors five in number, and the price of all their tickets $60. Dr. Brainard stands at the head of the faculty in this advertisement, and may be taken, I suppose, as the projector of the enterprize.

### *Multiplication of Schools.*

Every where that I go, our brethren are crying out against the multiplication of medical schools. But to what end? Who can arrest it? The medical school diathesis of the profession and the States, is like some morbid actions, 'self-limited,' and must be left to correct itself. It is of no use to be croaking about 'their increase,' but we may and should complain of under-bidding; and to see, as in the schools of Illinois, the number of Professors reduced to five, and the aggregate amount of their tickets to $60, is a cheapening that ought to be condemned. I fancy that the Professors would take exceptions to their brethren's reducing the price of their visits to twenty-five cents, or that of an amputation to 'fifteen shillings,' and with such feelings and opinions of professional propriety, they ought not to sell their lectures at a discount of 33⅓ per cent. If the young men of Illinois cannot afford the ordinary and established expenditure for medical education, they had better devote themselves to the cultivation of its millions of unenclosed and exuberantly fertile acres, and thus create the means of educating the next generation.

By the way, the *country* seems to me, in this State, to be the best locality for a physician. Illinois has no circulating, but a lively *locomotive* medium, which a physician might accumulate around him in abundance. As I was lately riding out with one of the oldest physicians in the State, he met a patient, who insisted on paying his bill in horses or fat cattle, and another took me to his farm, to show me, among other objects, the colts and calves which he had derived from the same source. In this way a physician might soon overspread a grazing farm with stock, and by its means replenish his stock of quinine when exhausted, which cannot always be done, with the whole amount of money received from those who consume it.

OTTOWA, September 19, 1844.

Spending half a day in Peru, I reached this place last evening. When at Bloomington and Peoria, I thought I saw evidence that

*Autumnal Fever*

was less prevalent than in the towns below, either in Missouri or Illinois. At Peru, its prevalence was still less, and here I find, as there, that the people are quite healthy. Thus I have traveled out of the region in which, *this year,* it is specially prevalent. Its northern limit lies a little above the 40th° of latitude. I find, however, that the

*June Flood*

prevailed here, and that the Illinois river, at the mouth of Fox river, where I am writing, rose higher than it was known by the 'oldest inhabitants,' (a memory of 12 or 15 years!) to have risen before, in the summer. That this rise has not been followed by as much sickness hereabouts as below, may, *perhaps,* be ascribed to a greater elevation, and less width of the 'river bottoms.'

Will you excuse me, if I again embellish a page with

*The Paroquet?*

The last flock which I saw was a little above the town of Hennepin, about the 41st° of latitude; and Dr. Lombard of this place tells me they do not come north of that limit. As far back as

1781, Mr. Jefferson attached importance to the northern migration of this beautiful bird, in estimating the relative heat of the climates east and west of the Alleghany—drawing from the observation an erroneous conclusion. The delightful Valley of the Illinois, seems at all times to have been the favorite haunt of the paroquet. I have seen more since I came into it, than in all other localities which I have visited this summer; and Father Marquette and M. Joliet, the first Europeans who ever traversed it—more than 170 years ago—saw them in great numbers. If you ask, what Ornithology has to do with medicine? I know of no more appropriate answer than this: the Romans, before going to battle, noticed the flight of birds, and Napoleon has compared a physician, contending against disease, to a General in the field—now if the flight of birds was useful in war, it may be so in physic. Certain, I am, that many flightier prescriptions have been made, than what the flight of birds would have suggested.

JOLIET, September 20, 1844.

I am now in latitude 41° 30′, nearly as far north as the southern end of Lake Michigan, and having finished my inquiries of the medical gentlemen of Joliet, I sit down at my chamber window, 9 P. M., serenaded by the rapids of the beautiful *Rivière des Plaines*, to continue, perhaps close, this philosophical epistle. The first matter to which I ask your attention, is,

*Juliet, versus Joliet!*

You will *Swiftly* exclaim—

"Strange such a difference should be,
'Twixt tweedledum and tweedledee"—

but don't be too fast. The joint discoverer and first navigator of the Mississippi, deserves at least a *nominal* monument, in some one of its thousand vales, and none more appropriate than that of the Illinois, through which he and his partner returned from their voyage of discovery, in 1617. It was to commemorate this daring adventure, through 1500 miles of perilous wilderness (now the home of more than 1500 doctors), that the proprietor of this town gave it the name of Joliet; which the people, with more gallantry than gratitude, have corrupted into Juliet. I call on both of you, as candidates for fame, to do all you can to restore to the intrepid pioneer the memorial which he is about to lose. Should you think this matter out of your line, I am sure you will not despise that which I am now about to present, under the head of

### *Triumphs of Mesmerism.*

A medical gentleman of this place informs me, that he can at any time relieve spinal irritation, by mesmerism; also, that it is one of the best means of preventing abortion which he has ever tried. He can mesmerize a nervous patient after lying down for the night, and she will sleep soundly till he awakes her, which he can do, by an effort of his will, transmitted from his own house. Still further, he can prevent the paroxysm of an intermittent fever, by mesmerizing his patient before the access of the chill, but the next paroxysm will come on. However, he has discovered (taking the hint from a work on homæopathy), that by producing an artificial chill during the intermission a radical cure is effected. This he accomplishes by producing mesmeric sleep, and then applying his finger to the tip of the patient's nose. Finally, the following case has lately occurred in his practice. A lady was subject to such obstinate constipation, that she passed fourteen days without an evacuation, when a drachm of calomel and six drops of castor oil produced only one discharge. Eight days after that, not having had another, he threw her into the mesmeric sleep, when mesmerizing a glass of water, he gave it to her as a solution of Epsom salts, the taste of which she admitted: he then made her hold half an ounce of calomel in her hand for about 30 minutes, when he awoke her. I do not recollect whether he mesmerized the calomel. When he called the next morning, he found that she had been purged to excess, and was profusely salivated, with swollen jaws and a mercurial breath. He then mesmerized her again, and putting a roll of brimstone (not mesmerized) into her hand, and applying another to her cheek, she was perfectly well in three days, and her costiveness has not since returned.

### *Consumption in Illinois.*

For the last fortnight, during which I have conversed with the physicians of seven towns between Jacksonville and Joliet, I have been assured, *nemine contradicente*, that tubercular consumption is one of the rarest diseases of that part of Illinois which lies between St. Louis and Chicago. Most of them have not seen an indigenous case, and all have known of immigrants in whom the progress of the disease, in its early stage, had been arrested or retarded. As many of our readers may be consulted by their patients, concerning the effect of the climate of this region on those inclined to phthisis, I have thought it proper to anticipate a future publication by this brief notice.

Very respectfully, your
Obedient servant,
D.

